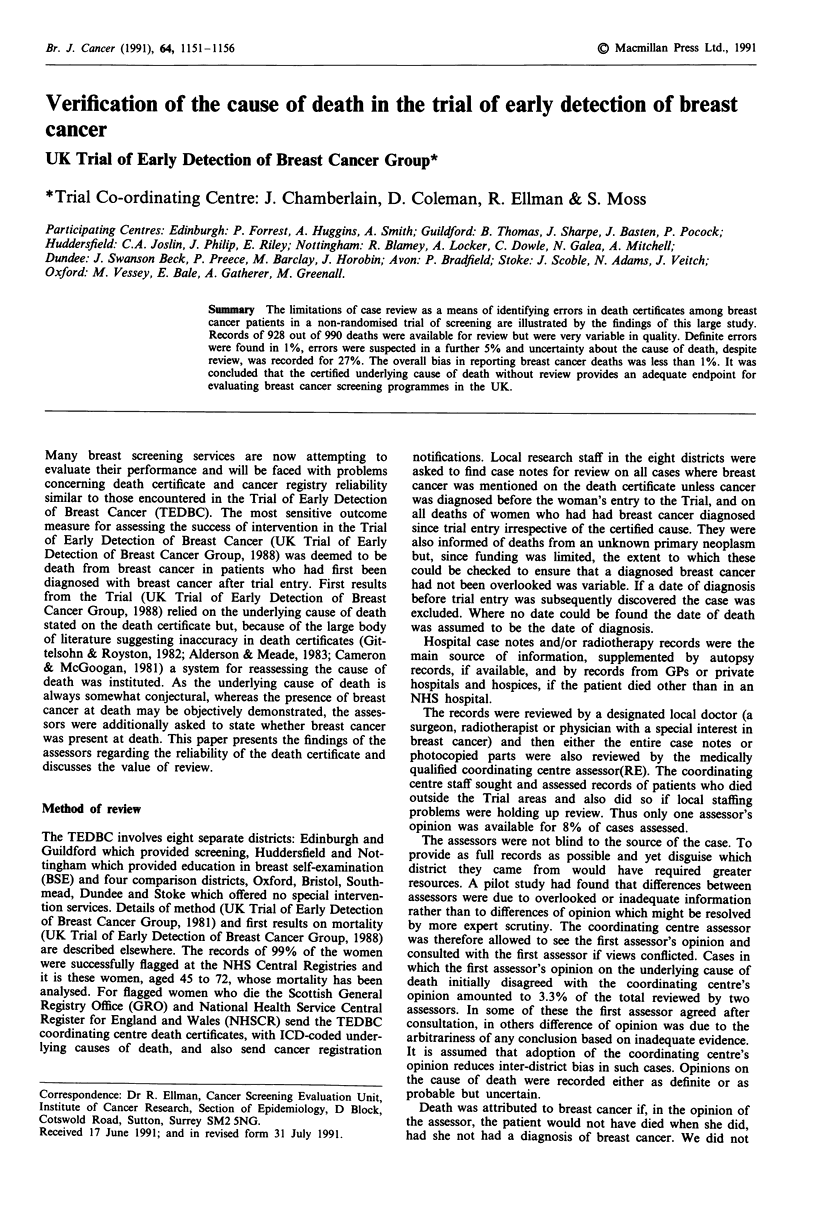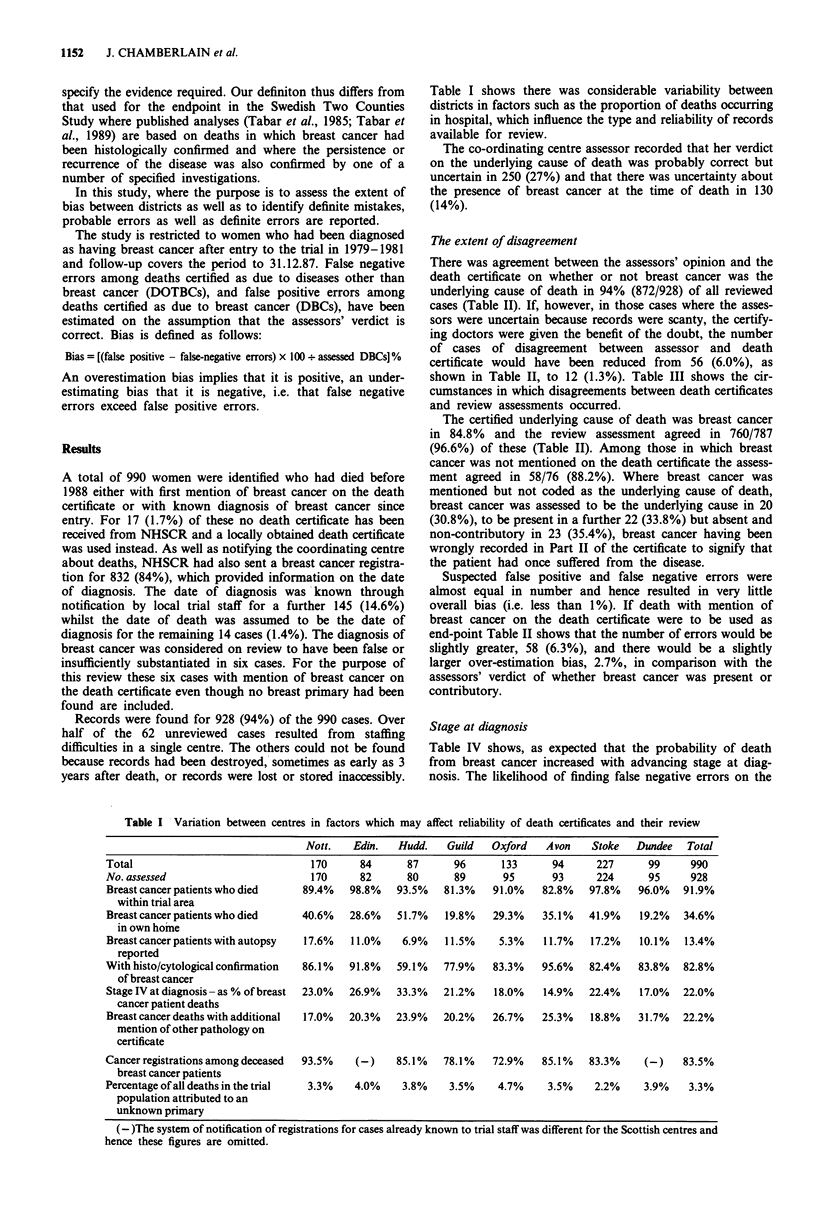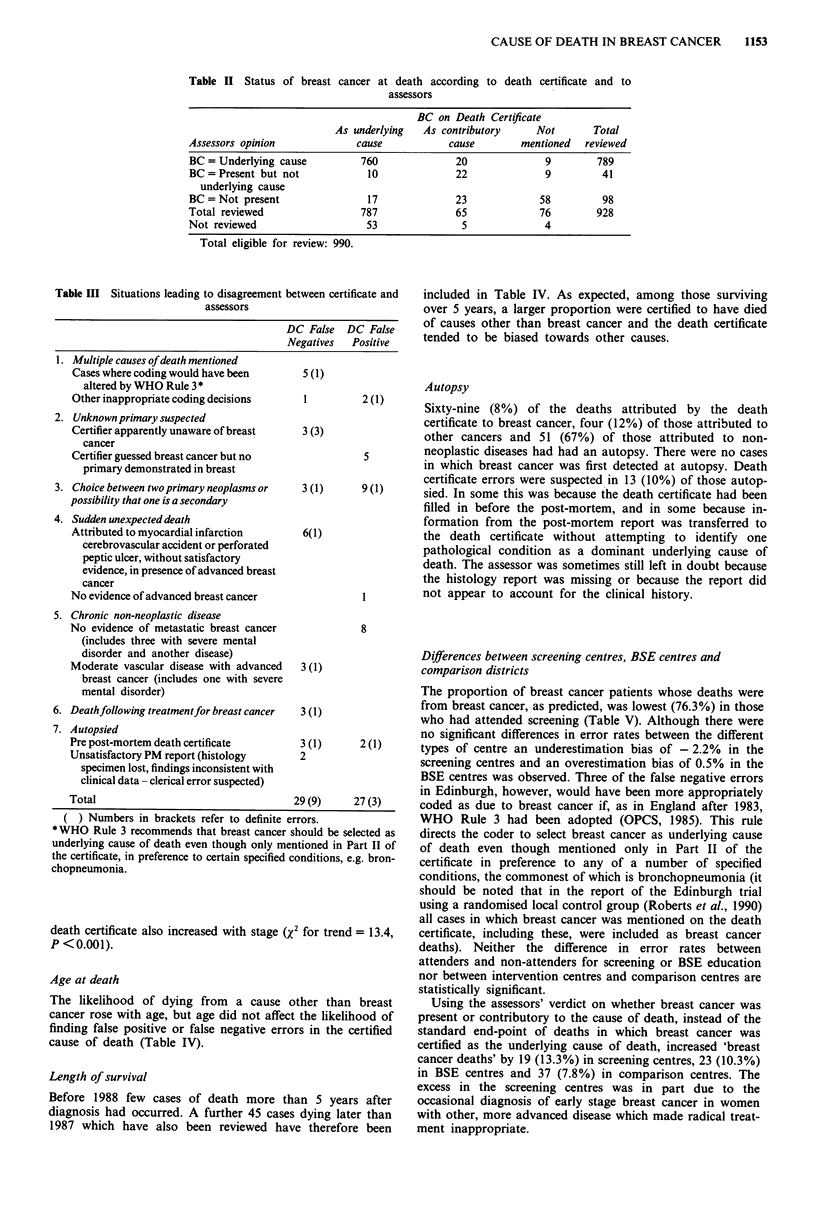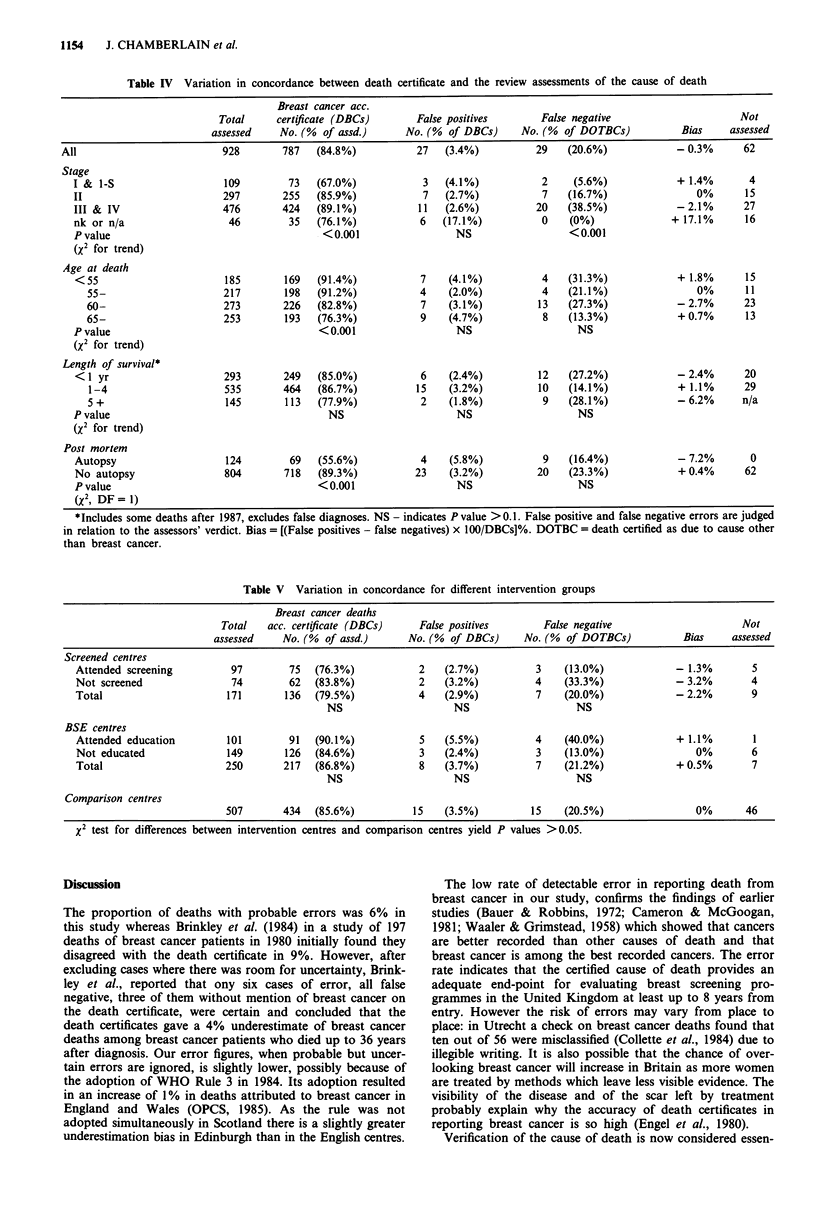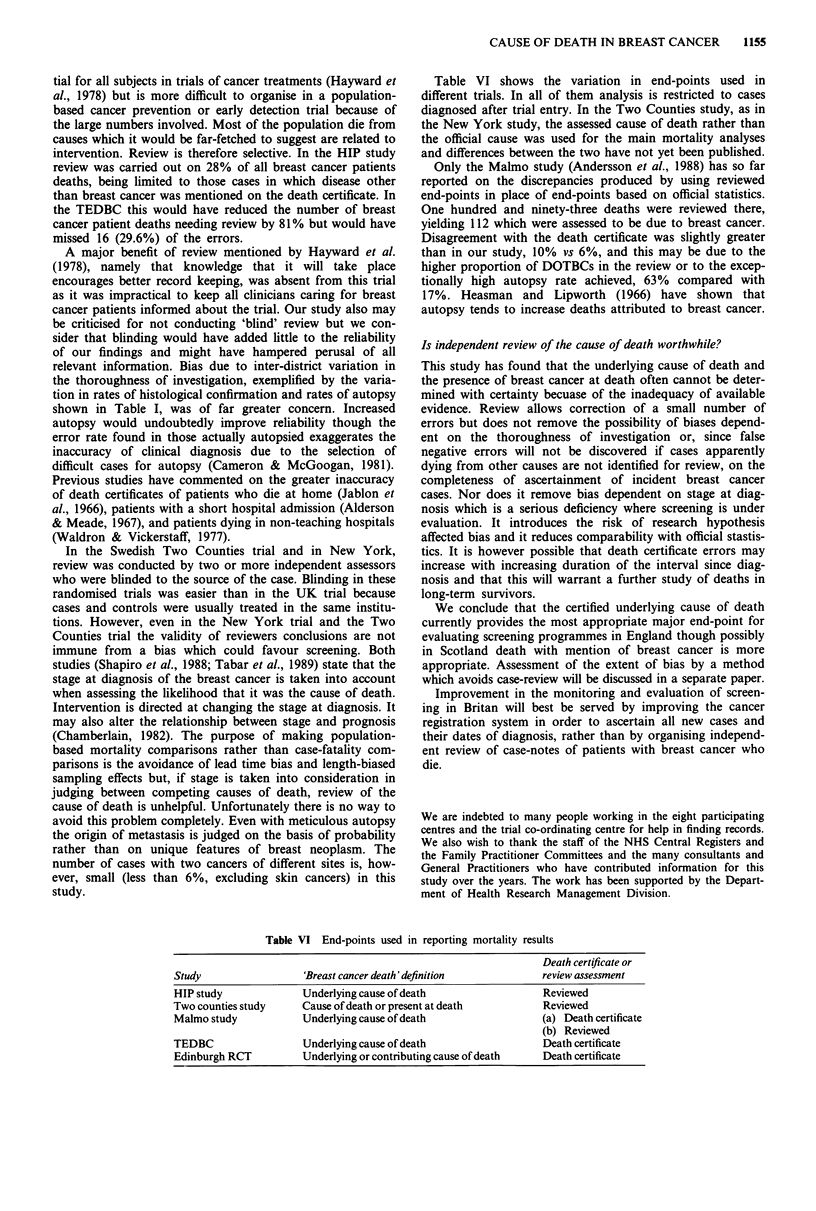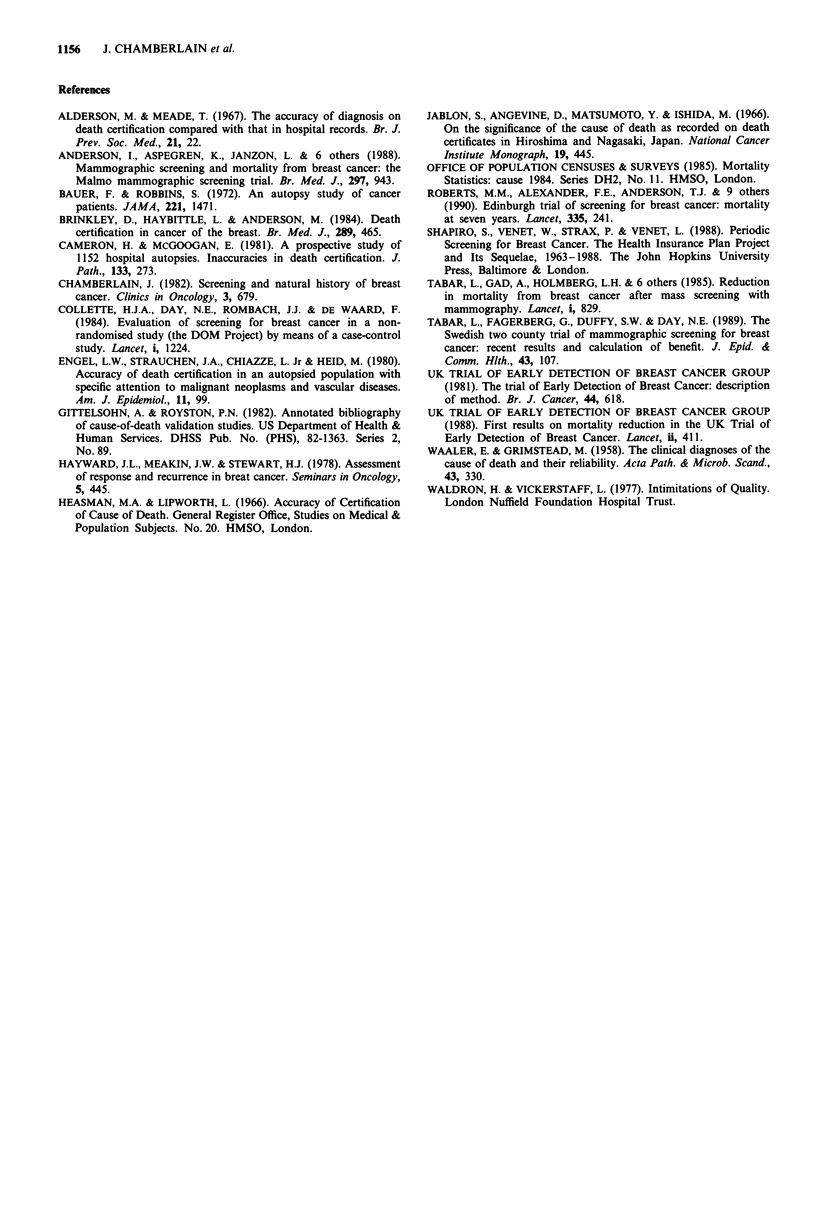# Verification of the cause of death in the trial of early detection of breast cancer. UK Trial of Early Detection of Breast Cancer Group. Trial Co-ordinating Centre.

**DOI:** 10.1038/bjc.1991.480

**Published:** 1991-12

**Authors:** J. Chamberlain, D. Coleman, R. Ellman, S. Moss

**Affiliations:** Institute of Cancer Research, Section of Epidemiology, Sutton, Surrey.

## Abstract

The limitations of case review as a means of identifying errors in death certificates among breast cancer patients in a non-randomised trial of screening are illustrated by the findings of this large study. Records of 928 out of 990 deaths were available for review but were very variable in quality. Definite errors were found in 1%, errors were suspected in a further 5% and uncertainty about the cause of death, despite review, was recorded for 27%. The overall bias in reporting breast cancer deaths was less than 1%. It was concluded that the certified underlying cause of death without review provides an adequate endpoint for evaluating breast cancer screening programmes in the UK.


Br. J. Cancer (1991), 64, 1151–1156 © Macmillan Press Ltd., 1991

### Verification of the cause of death in the trial of early detection of breast cancer

**UK Trial of Early Detection of Breast Cancer Group***

*Trial Co-ordinating Centre: J. Chamberlain, D. Coleman, R. Ellman & S. Moss

*Participating Centres: Edinburgh: P. Forrest, A. Huggins, A. Smith; Guildford: B. Thomas, J. Sharpe, J. Basten, P. Pocock; Huddersfield: C.A. Joslin, J. Philip, E. Riley; Nottingham: R. Blamey, A. Locker, C. Dowle, N. Galea, A. Mitchell; Dundee: J. Swanson Beck, P. Preece, M. Barclay, J. Horobin; Avon: P. Bradfield; Stoke: J. Scoble, N. Adams, J. Veitch; Oxford: M. Vessey, E. Bale, A. Gatherer, M. Greenall.*

**Summary** The limitations of case review as a means of identifying errors in death certificates among breast cancer patients in a non-randomised trial of screening are illustrated by the findings of this large study. Records of 928 out of 990 deaths were available for review but were very variable in quality. Definite errors were found in 1%, errors were suspected in a further 5% and uncertainty about the cause of death, despite review, was recorded for 27%. The overall bias in reporting breast cancer deaths was less than 1%. It was concluded that the certified underlying cause of death without review provides an adequate endpoint for evaluating breast cancer screening programmes in the UK.

Many breast screening services are now attempting to evaluate their performance and will be faced with problems concerning death certificate and cancer registry reliability similar to those encountered in the Trial of Early Detection of Breast Cancer (TEDBC). The most sensitive outcome measure for assessing the success of intervention in the Trial of Early Detection of Breast Cancer (UK Trial of Early Detection of Breast Cancer Group, 1988) was deemed to be death from breast cancer in patients who had first been diagnosed with breast cancer after trial entry. First results from the Trial (UK Trial of Early Detection of Breast Cancer Group, 1988) relied on the underlying cause of death stated on the death certificate but, because of the large body of literature suggesting inaccuracy in death certificates (Gittelsohn & Royston, 1982; Alderson & Meade, 1983; Cameron & McGoogan, 1981) a system for reassessing the cause of death was instituted. As the underlying cause of death is always somewhat conjectural, whereas the presence of breast cancer at death may be objectively demonstrated, the assessors were additionally asked to state whether breast cancer was present at death. This paper presents the findings of the assessors regarding the reliability of the death certificate and discusses the value of review.

### Method of review

The TEDBC involves eight separate districts: Edinburgh and Guildford which provided screening, Huddersfield and Nottingham which provided education in breast self-examination (BSE) and four comparison districts, Oxford, Bristol, Southmead, Dundee and Stoke which offered no special intervention services. Details of method (UK Trial of Early Detection of Breast Cancer Group, 1981) and first results on mortality (UK Trial of Early Detection of Breast Cancer Group, 1988) are described elsewhere. The records of 99% of the women were successfully flagged at the NHS Central Registries and it is these women, aged 45 to 72, whose mortality has been analysed. For flagged women who die the Scottish General Registry Office (GRO) and National Health Service Central Register for England and Wales (NHSCR) send the TEDBC coordinating centre death certificates, with ICD-coded underlying causes of death, and also send cancer registration notifications. Local research staff in the eight districts were asked to find case notes for review on all cases where breast cancer was mentioned on the death certificate unless cancer was diagnosed before the woman's entry to the Trial, and on all deaths of women who had had breast cancer diagnosed since trial entry irrespective of the certified cause. They were also informed of deaths from an unknown primary neoplasm but, since funding was limited, the extent to which these could be checked to ensure that a diagnosed breast cancer had not been overlooked was variable. If a date of diagnosis before trial entry was subsequently discovered the case was excluded. Where no date could be found the date of death was assumed to be the date of diagnosis.

Hospital case notes and/or radiotherapy records were the main source of information, supplemented by autopsy records, if available, and by records from GPs or private hospitals and hospices, if the patient died other than in an NHS hospital.

The records were reviewed by a designated local doctor (a surgeon, radiotherapist or physician with a special interest in breast cancer) and then either the entire case notes or photocopied parts were also reviewed by the medically qualified coordinating centre assessor(RE). The coordinating centre staff sought and assessed records of patients who died outside the Trial areas and also did so if local staffing problems were holding up review. Thus only one assessor's opinion was available for 8% of cases assessed.

The assessors were not blind to the source of the case. To provide as full records as possible and yet disguise which district they came from would have required greater resources. A pilot study had found that differences between assessors were due to overlooked or inadequate information rather than to differences of opinion which might be resolved by more expert scrutiny. The coordinating centre assessor was therefore allowed to see the first assessor's opinion and consulted with the first assessor if views conflicted. Cases in which the first assessor's opinion on the underlying cause of death initially disagreed with the coordinating centre's opinion amounted to 3.3% of the total reviewed by two assessors. In some of these the first assessor agreed after consultation, in others difference of opinion was due to the arbitrariness of any conclusion based on inadequate evidence. It is assumed that adoption of the coordinating centre's opinion reduces inter-district bias in such cases. Opinions on the cause of death were recorded either as definite or as probable but uncertain.

Death was attributed to breast cancer if, in the opinion of the assessor, the patient would not have died when she did, had she not had a diagnosis of breast cancer. We did not

Correspondence: Dr R. Ellman, Cancer Screening Evaluation Unit, Institute of Cancer Research, Section of Epidemiology, D Block, Cotswold Road, Sutton, Surrey SM2 5NG.
Received 17 June 1991; and in revised form 31 July 1991.

specify the evidence required. Our definiton thus differs from that used for the endpoint in the Swedish Two Counties Study where published analyses (Tabar *et al.*, 1985; Tabar *et al.*, 1989) are based on deaths in which breast cancer had been histologically confirmed and where the persistence or recurrence of the disease was also confirmed by one of a number of specified investigations.

In this study, where the purpose is to assess the extent of bias between districts as well as to identify definite mistakes, probable errors as well as definite errors are reported.

The study is restricted to women who had been diagnosed as having breast cancer after entry to the trial in 1979–1981 and follow-up covers the period to 31.12.87. False negative errors among deaths certified as due to diseases other than breast cancer (DOTBCs), and false positive errors among deaths certified as due to breast cancer (DBCs), have been estimated on the assumption that the assessors' verdict is correct. Bias is defined as follows:

Bias = [(false positive − false-negative errors) × 100 ÷ assessed DBCs] %

An overestimation bias implies that it is positive, an underestimating bias that it is negative, i.e. that false negative errors exceed false positive errors.

### Results

A total of 990 women were identified who had died before 1988 either with first mention of breast cancer on the death certificate or with known diagnosis of breast cancer since entry. For 17 (1.7%) of these no death certificate has been received from NHSCR and a locally obtained death certificate was used instead. As well as notifying the coordinating centre about deaths, NHSCR had also sent a breast cancer registration for 832 (84%), which provided information on the date of diagnosis. The date of diagnosis was known through notification by local trial staff for a further 145 (14.6%) whilst the date of death was assumed to be the date of diagnosis for the remaining 14 cases (1.4%). The diagnosis of breast cancer was considered on review to have been false or insufficiently substantiated in six cases. For the purpose of this review these six cases with mention of breast cancer on the death certificate even though no breast primary had been found are included.

Records were found for 928 (94%) of the 990 cases. Over half of the 62 unreviewed cases resulted from staffing difficulties in a single centre. The others could not be found because records had been destroyed, sometimes as early as 3 years after death, or records were lost or stored inaccessibly. Table I shows there was considerable variability between districts in factors such as the proportion of deaths occurring in hospital, which influence the type and reliability of records available for review.

The co-ordinating centre assessor recorded that her verdict on the underlying cause of death was probably correct but uncertain in 250 (27%) and that there was uncertainty about the presence of breast cancer at the time of death in 130 (14%).

### *The extent of disagreement*

There was agreement between the assessors' opinion and the death certificate on whether or not breast cancer was the underlying cause of death in 94% (872/928) of all reviewed cases (Table II). If, however, in those cases where the assessors were uncertain because records were scanty, the certifying doctors were given the benefit of the doubt, the number of cases of disagreement between assessor and death certificate would have been reduced from 56 (6.0%), as shown in Table II, to 12 (1.3%). Table III shows the circumstances in which disagreements between death certificates and review assessments occurred.

The certified underlying cause of death was breast cancer in 84.8% and the review assessment agreed in 760/787 (96.6%) of these (Table II). Among those in which breast cancer was not mentioned on the death certificate the assessment agreed in 58/76 (88.2%). Where breast cancer was mentioned but not coded as the underlying cause of death, breast cancer was assessed to be the underlying cause in 20 (30.8%), to be present in a further 22 (33.8%) but absent and non-contributory in 23 (35.4%), breast cancer having been wrongly recorded in Part II of the certificate to signify that the patient had once suffered from the disease.

Suspected false positive and false negative errors were almost equal in number and hence resulted in very little overall bias (i.e. less than 1%). If death with mention of breast cancer on the death certificate were to be used as end-point Table II shows that the number of errors would be slightly greater, 58 (6.3%), and there would be a slightly larger over-estimation bias, 2.7%, in comparison with the assessors' verdict of whether breast cancer was present or contributory.

### *Stage at diagnosis*

Table IV shows, as expected that the probability of death from breast cancer increased with advancing stage at diagnosis. The likelihood of finding false negative errors on the

**Table I** Variation between centres in factors which may affect reliability of death certificates and their review

| | *Nott.* | *Edin.* | *Hudd.* | *Guild* | *Oxford* | *Avon* | *Stoke* | *Dundee* | *Total* |
|---|---|---|---|---|---|---|---|---|---|
| Total | 170 | 84 | 87 | 96 | 133 | 94 | 227 | 99 | 990 |
| *No. assessed* | 170 | 82 | 80 | 89 | 95 | 93 | 224 | 95 | 928 |
| Breast cancer patients who died within trial area | 89.4% | 98.8% | 93.5% | 81.3% | 91.0% | 82.8% | 97.8% | 96.0% | 91.9% |
| Breast cancer patients who died in own home | 40.6% | 28.6% | 51.7% | 19.8% | 29.3% | 35.1% | 41.9% | 19.2% | 34.6% |
| Breast cancer patients with autopsy reported | 17.6% | 11.0% | 6.9% | 11.5% | 5.3% | 11.7% | 17.2% | 10.1% | 13.4% |
| With histo/cytological confirmation of breast cancer | 86.1% | 91.8% | 59.1% | 77.9% | 83.3% | 95.6% | 82.4% | 83.8% | 82.8% |
| Stage IV at diagnosis – as % of breast cancer patient deaths | 23.0% | 26.9% | 33.3% | 21.2% | 18.0% | 14.9% | 22.4% | 17.0% | 22.0% |
| Breast cancer deaths with additional mention of other pathology on certificate | 17.0% | 20.3% | 23.9% | 20.2% | 26.7% | 25.3% | 18.8% | 31.7% | 22.2% |
| Cancer registrations among deceased breast cancer patients | 93.5% | (−) | 85.1% | 78.1% | 72.9% | 85.1% | 83.3% | (−) | 83.5% |
| Percentage of all deaths in the trial population attributed to an unknown primary | 3.3% | 4.0% | 3.8% | 3.5% | 4.7% | 3.5% | 2.2% | 3.9% | 3.3% |

(−)The system of notification of registrations for cases already known to trial staff was different for the Scottish centres and hence these figures are omitted.

**Table II** Status of breast cancer at death according to death certificate and to assessors

| | BC on Death Certificate | | | |
|---|---|---|---|---|
| *Assessors opinion* | *As underlying cause* | *As contributory cause* | *Not mentioned* | *Total reviewed* |
| BC = Underlying cause | 760 | 20 | 9 | 789 |
| BC = Present but not underlying cause | 10 | 22 | 9 | 41 |
| BC = Not present | 17 | 23 | 58 | 98 |
| Total reviewed | 787 | 65 | 76 | 928 |
| Not reviewed | 53 | 5 | 4 | |

Total eligible for review: 990.

**Table III** Situations leading to disagreement between certificate and assessors

| | *DC False Negatives* | *DC False Positive* |
|---|---|---|
| 1. *Multiple causes of death mentioned* | | |
| Cases where coding would have been altered by WHO Rule 3* | 5 (1) | |
| Other inappropriate coding decisions | 1 | 2 (1) |
| 2. *Unknown primary suspected* | | |
| Certifier apparently unaware of breast cancer | 3 (3) | |
| Certifier guessed breast cancer but no primary demonstrated in breast | | 5 |
| 3. *Choice between two primary neoplasms or possibility that one is a secondary* | 3 (1) | 9 (1) |
| 4. *Sudden unexpected death* | | |
| Attributed to myocardial infarction cerebrovascular accident or perforated peptic ulcer, without satisfactory evidence, in presence of advanced breast cancer | 6(1) | |
| No evidence of advanced breast cancer | | 1 |
| 5. *Chronic non-neoplastic disease* | | |
| No evidence of metastatic breast cancer (includes three with severe mental disorder and another disease) | | 8 |
| Moderate vascular disease with advanced breast cancer (includes one with severe mental disorder) | 3 (1) | |
| 6. *Death following treatment for breast cancer* | 3 (1) | |
| 7. *Autopsied* | | |
| Pre post-mortem death certificate | 3 (1) | 2 (1) |
| Unsatisfactory PM report (histology specimen lost, findings inconsistent with clinical data – clerical error suspected) | 2 | |
| Total | 29 (9) | 27 (3) |

( ) Numbers in brackets refer to definite errors.
*WHO Rule 3 recommends that breast cancer should be selected as underlying cause of death even though only mentioned in Part II of the certificate, in preference to certain specified conditions, e.g. bronchopneumonia.

death certificate also increased with stage ($\chi^2$ for trend = 13.4, $P < 0.001$).

### *Age at death*

The likelihood of dying from a cause other than breast cancer rose with age, but age did not affect the likelihood of finding false positive or false negative errors in the certified cause of death (Table IV).

### *Length of survival*

Before 1988 few cases of death more than 5 years after diagnosis had occurred. A further 45 cases dying later than 1987 which have also been reviewed have therefore been included in Table IV. As expected, among those surviving over 5 years, a larger proportion were certified to have died of causes other than breast cancer and the death certificate tended to be biased towards other causes.

### *Autopsy*

Sixty-nine (8%) of the deaths attributed by the death certificate to breast cancer, four (12%) of those attributed to other cancers and 51 (67%) of those attributed to non-neoplastic diseases had had an autopsy. There were no cases in which breast cancer was first detected at autopsy. Death certificate errors were suspected in 13 (10%) of those autopsied. In some this was because the death certificate had been filled in before the post-mortem, and in some because information from the post-mortem report was transferred to the death certificate without attempting to identify one pathological condition as a dominant underlying cause of death. The assessor was sometimes still left in doubt because the histology report was missing or because the report did not appear to account for the clinical history.

### *Differences between screening centres, BSE centres and comparison districts*

The proportion of breast cancer patients whose deaths were from breast cancer, as predicted, was lowest (76.3%) in those who had attended screening (Table V). Although there were no significant differences in error rates between the different types of centre an underestimation bias of − 2.2% in the screening centres and an overestimation bias of 0.5% in the BSE centres was observed. Three of the false negative errors in Edinburgh, however, would have been more appropriately coded as due to breast cancer if, as in England after 1983, WHO Rule 3 had been adopted (OPCS, 1985). This rule directs the coder to select breast cancer as underlying cause of death even though mentioned only in Part II of the certificate in preference to any of a number of specified conditions, the commonest of which is bronchopneumonia (it should be noted that in the report of the Edinburgh trial using a randomised local control group (Roberts *et al*., 1990) all cases in which breast cancer was mentioned on the death certificate, including these, were included as breast cancer deaths). Neither the difference in error rates between attenders and non-attenders for screening or BSE education nor between intervention centres and comparison centres are statistically significant.

Using the assessors' verdict on whether breast cancer was present or contributory to the cause of death, instead of the standard end-point of deaths in which breast cancer was certified as the underlying cause of death, increased 'breast cancer deaths' by 19 (13.3%) in screening centres, 23 (10.3%) in BSE centres and 37 (7.8%) in comparison centres. The excess in the screening centres was in part due to the occasional diagnosis of early stage breast cancer in women with other, more advanced disease which made radical treatment inappropriate.

**Table IV** Variation in concordance between death certificate and the review assessments of the cause of death

| | Total assessed | Breast cancer acc. certificate (DBCs) No. (% of assd.) | | False positives No. (% of DBCs) | | False negative No. (% of DOTBCs) | | Bias | Not assessed |
|---|---|---|---|---|---|---|---|---|---|
| All | 928 | 787 | (84.8%) | 27 | (3.4%) | 29 | (20.6%) | − 0.3% | 62 |
| *Stage* | | | | | | | | | |
| I & 1-S | 109 | 73 | (67.0%) | 3 | (4.1%) | 2 | (5.6%) | + 1.4% | 4 |
| II | 297 | 255 | (85.9%) | 7 | (2.7%) | 7 | (16.7%) | 0% | 15 |
| III & IV | 476 | 424 | (89.1%) | 11 | (2.6%) | 20 | (38.5%) | − 2.1% | 27 |
| nk or n/a | 46 | 35 | (76.1%) | 6 | (17.1%) | 0 | (0%) | + 17.1% | 16 |
| *P* value ($\chi^2$ for trend) | | | <0.001 | | NS | | <0.001 | | |
| *Age at death* | | | | | | | | | |
| <55 | 185 | 169 | (91.4%) | 7 | (4.1%) | 4 | (31.3%) | + 1.8% | 15 |
| 55– | 217 | 198 | (91.2%) | 4 | (2.0%) | 4 | (21.1%) | 0% | 11 |
| 60– | 273 | 226 | (82.8%) | 7 | (3.1%) | 13 | (27.3%) | − 2.7% | 23 |
| 65– | 253 | 193 | (76.3%) | 9 | (4.7%) | 8 | (13.3%) | + 0.7% | 13 |
| *P* value ($\chi^2$ for trend) | | | <0.001 | | NS | | NS | | |
| *Length of survival** | | | | | | | | | |
| <1 yr | 293 | 249 | (85.0%) | 6 | (2.4%) | 12 | (27.2%) | − 2.4% | 20 |
| 1–4 | 535 | 464 | (86.7%) | 15 | (3.2%) | 10 | (14.1%) | + 1.1% | 29 |
| 5+ | 145 | 113 | (77.9%) | 2 | (1.8%) | 9 | (28.1%) | − 6.2% | n/a |
| *P* value ($\chi^2$ for trend) | | | NS | | NS | | NS | | |
| *Post mortem* | | | | | | | | | |
| Autopsy | 124 | 69 | (55.6%) | 4 | (5.8%) | 9 | (16.4%) | − 7.2% | 0 |
| No autopsy | 804 | 718 | (89.3%) | 23 | (3.2%) | 20 | (23.3%) | + 0.4% | 62 |
| *P* value ($\chi^2$, DF = 1) | | | <0.001 | | NS | | NS | | |

*Includes some deaths after 1987, excludes false diagnoses. NS – indicates *P* value >0.1. False positive and false negative errors are judged in relation to the assessors' verdict. Bias = [(False positives – false negatives) x 100/DBCs]%. DOTBC = death certified as due to cause other than breast cancer.

**Table V** Variation in concordance for different intervention groups

| | Total assessed | Breast cancer deaths acc. certificate (DBCs) No. (% of assd.) | | False positives No. (% of DBCs) | | False negative No. (% of DOTBCs) | | Bias | Not assessed |
|---|---|---|---|---|---|---|---|---|---|
| *Screened centres* | | | | | | | | | |
| Attended screening | 97 | 75 | (76.3%) | 2 | (2.7%) | 3 | (13.0%) | − 1.3% | 5 |
| Not screened | 74 | 62 | (83.8%) | 2 | (3.2%) | 4 | (33.3%) | − 3.2% | 4 |
| Total | 171 | 136 | (79.5%) | 4 | (2.9%) | 7 | (20.0%) | − 2.2% | 9 |
| | | | NS | | NS | | NS | | |
| *BSE centres* | | | | | | | | | |
| Attended education | 101 | 91 | (90.1%) | 5 | (5.5%) | 4 | (40.0%) | + 1.1% | 1 |
| Not educated | 149 | 126 | (84.6%) | 3 | (2.4%) | 3 | (13.0%) | 0% | 6 |
| Total | 250 | 217 | (86.8%) | 8 | (3.7%) | 7 | (21.2%) | + 0.5% | 7 |
| | | | NS | | NS | | NS | | |
| *Comparison centres* | | | | | | | | | |
| | 507 | 434 | (85.6%) | 15 | (3.5%) | 15 | (20.5%) | 0% | 46 |

$\chi^2$ test for differences between intervention centres and comparison centres yield *P* values >0.05.

### Discussion

The proportion of deaths with probable errors was 6% in this study whereas Brinkley *et al.* (1984) in a study of 197 deaths of breast cancer patients in 1980 initially found they disagreed with the death certificate in 9%. However, after excluding cases where there was room for uncertainty, Brinkley *et al.*, reported that ony six cases of error, all false negative, three of them without mention of breast cancer on the death certificate, were certain and concluded that the death certificates gave a 4% underestimate of breast cancer deaths among breast cancer patients who died up to 36 years after diagnosis. Our error figures, when probable but uncertain errors are ignored, is slightly lower, possibly because of the adoption of WHO Rule 3 in 1984. Its adoption resulted in an increase of 1% in deaths attributed to breast cancer in England and Wales (OPCS, 1985). As the rule was not adopted simultaneously in Scotland there is a slightly greater underestimation bias in Edinburgh than in the English centres.

The low rate of detectable error in reporting death from breast cancer in our study, confirms the findings of earlier studies (Bauer & Robbins, 1972; Cameron & McGoogan, 1981; Waaler & Grimstead, 1958) which showed that cancers are better recorded than other causes of death and that breast cancer is among the best recorded cancers. The error rate indicates that the certified cause of death provides an adequate end-point for evaluating breast screening programmes in the United Kingdom at least up to 8 years from entry. However the risk of errors may vary from place to place: in Utrecht a check on breast cancer deaths found that ten out of 56 were misclassified (Collette *et al.*, 1984) due to illegible writing. It is also possible that the chance of overlooking breast cancer will increase in Britain as more women are treated by methods which leave less visible evidence. The visibility of the disease and of the scar left by treatment probably explain why the accuracy of death certificates in reporting breast cancer is so high (Engel *et al.*, 1980).

Verification of the cause of death is now considered essential for all subjects in trials of cancer treatments (Hayward *et al.*, 1978) but is more difficult to organise in a population-based cancer prevention or early detection trial because of the large numbers involved. Most of the population die from causes which it would be far-fetched to suggest are related to intervention. Review is therefore selective. In the HIP study review was carried out on 28% of all breast cancer patients deaths, being limited to those cases in which disease other than breast cancer was mentioned on the death certificate. In the TEDBC this would have reduced the number of breast cancer patient deaths needing review by 81% but would have missed 16 (29.6%) of the errors.

A major benefit of review mentioned by Hayward *et al.* (1978), namely that knowledge that it will take place encourages better record keeping, was absent from this trial as it was impractical to keep all clinicians caring for breast cancer patients informed about the trial. Our study also may be criticised for not conducting 'blind' review but we consider that blinding would have added little to the reliability of our findings and might have hampered perusal of all relevant information. Bias due to inter-district variation in the thoroughness of investigation, exemplified by the variation in rates of histological confirmation and rates of autopsy shown in Table I, was of far greater concern. Increased autopsy would undoubtedly improve reliability though the error rate found in those actually autopsied exaggerates the inaccuracy of clinical diagnosis due to the selection of difficult cases for autopsy (Cameron & McGoogan, 1981). Previous studies have commented on the greater inaccuracy of death certificates of patients who die at home (Jablon *et al.*, 1966), patients with a short hospital admission (Alderson & Meade, 1967), and patients dying in non-teaching hospitals (Waldron & Vickerstaff, 1977).

In the Swedish Two Counties trial and in New York, review was conducted by two or more independent assessors who were blinded to the source of the case. Blinding in these randomised trials was easier than in the UK trial because cases and controls were usually treated in the same institutions. However, even in the New York trial and the Two Counties trial the validity of reviewers conclusions are not immune from a bias which could favour screening. Both studies (Shapiro *et al.*, 1988; Tabar *et al.*, 1989) state that the stage at diagnosis of the breast cancer is taken into account when assessing the likelihood that it was the cause of death. Intervention is directed at changing the stage at diagnosis. It may also alter the relationship between stage and prognosis (Chamberlain, 1982). The purpose of making population-based mortality comparisons rather than case-fatality comparisons is the avoidance of lead time bias and length-biased sampling effects but, if stage is taken into consideration in judging between competing causes of death, review of the cause of death is unhelpful. Unfortunately there is no way to avoid this problem completely. Even with meticulous autopsy the origin of metastasis is judged on the basis of probability rather than on unique features of breast neoplasm. The number of cases with two cancers of different sites is, however, small (less than 6%, excluding skin cancers) in this study.

Table VI shows the variation in end-points used in different trials. In all of them analysis is restricted to cases diagnosed after trial entry. In the Two Counties study, as in the New York study, the assessed cause of death rather than the official cause was used for the main mortality analyses and differences between the two have not yet been published.

Only the Malmo study (Andersson *et al.*, 1988) has so far reported on the discrepancies produced by using reviewed end-points in place of end-points based on official statistics. One hundred and ninety-three deaths were reviewed there, yielding 112 which were assessed to be due to breast cancer. Disagreement with the death certificate was slightly greater than in our study, 10% *vs* 6%, and this may be due to the higher proportion of DOTBCs in the review or to the exceptionally high autopsy rate achieved, 63% compared with 17%. Heasman and Lipworth (1966) have shown that autopsy tends to increase deaths attributed to breast cancer.

*Is independent review of the cause of death worthwhile?*

This study has found that the underlying cause of death and the presence of breast cancer at death often cannot be determined with certainty becuase of the inadequacy of available evidence. Review allows correction of a small number of errors but does not remove the possibility of biases dependent on the thoroughness of investigation or, since false negative errors will not be discovered if cases apparently dying from other causes are not identified for review, on the completeness of ascertainment of incident breast cancer cases. Nor does it remove bias dependent on stage at diagnosis which is a serious deficiency where screening is under evaluation. It introduces the risk of research hypothesis affected bias and it reduces comparability with official stastistics. It is however possible that death certificate errors may increase with increasing duration of the interval since diagnosis and that this will warrant a further study of deaths in long-term survivors.

We conclude that the certified underlying cause of death currently provides the most appropriate major end-point for evaluating screening programmes in England though possibly in Scotland death with mention of breast cancer is more appropriate. Assessment of the extent of bias by a method which avoids case-review will be discussed in a separate paper.

Improvement in the monitoring and evaluation of screening in Britan will best be served by improving the cancer registration system in order to ascertain all new cases and their dates of diagnosis, rather than by organising independent review of case-notes of patients with breast cancer who die.

We are indebted to many people working in the eight participating centres and the trial co-ordinating centre for help in finding records. We also wish to thank the staff of the NHS Central Registers and the Family Practitioner Committees and the many consultants and General Practitioners who have contributed information for this study over the years. The work has been supported by the Department of Health Research Management Division.

**Table VI** End-points used in reporting mortality results

| *Study* | *'Breast cancer death' definition* | *Death certificate or review assessment* |
|---|---|---|
| HIP study | Underlying cause of death | Reviewed |
| Two counties study | Cause of death or present at death | Reviewed |
| Malmo study | Underlying cause of death | (a) Death certificate<br>(b) Reviewed |
| TEDBC | Underlying cause of death | Death certificate |
| Edinburgh RCT | Underlying or contributing cause of death | Death certificate |

### References

ALDERSON, M. & MEADE, T. (1967). The accuracy of diagnosis on death certification compared with that in hospital records. *Br. J. Prev. Soc. Med.*, **21**, 22.

ANDERSON, I., ASPEGREN, K., JANZON, L. & 6 others (1988). Mammographic screening and mortality from breast cancer: the Malmo mammographic screening trial. *Br. Med. J.*, **297**, 943.

BAUER, F. & ROBBINS, S. (1972). An autopsy study of cancer patients. *JAMA*, **221**, 1471.

BRINKLEY, D., HAYBITTLE, L. & ANDERSON, M. (1984). Death certification in cancer of the breast. *Br. Med. J.*, **289**, 465.

CAMERON, H. & MCGOOGAN, E. (1981). A prospective study of 1152 hospital autopsies. Inaccuracies in death certification. *J. Path.*, **133**, 273.

CHAMBERLAIN, J. (1982). Screening and natural history of breast cancer. *Clinics in Oncology*, **3**, 679.

COLLETTE, H.J.A., DAY, N.E., ROMBACH, J.J. & DE WAARD, F. (1984). Evaluation of screening for breast cancer in a non-randomised study (the DOM Project) by means of a case-control study. *Lancet*, **i**, 1224.

ENGEL, L.W., STRAUCHEN, J.A., CHIAZZE, L. Jr & HEID, M. (1980). Accuracy of death certification in an autopsied population with specific attention to malignant neoplasms and vascular diseases. *Am. J. Epidemiol.*, **11**, 99.

GITTELSOHN, A. & ROYSTON, P.N. (1982). Annotated bibliography of cause-of-death validation studies. US Department of Health & Human Services. DHSS Pub. No. (PHS), 82-1363. Series 2, No. 89.

HAYWARD, J.L., MEAKIN, J.W. & STEWART, H.J. (1978). Assessment of response and recurrence in breat cancer. *Seminars in Oncology*, **5**, 445.

HEASMAN, M.A. & LIPWORTH, L. (1966). Accuracy of Certification of Cause of Death. General Register Office, Studies on Medical & Population Subjects. No. 20. HMSO, London.

JABLON, S., ANGEVINE, D., MATSUMOTO, Y. & ISHIDA, M. (1966). On the significance of the cause of death as recorded on death certificates in Hiroshima and Nagasaki, Japan. *National Cancer Institute Monograph*, **19**, 445.

OFFICE OF POPULATION CENSUSES & SURVEYS (1985). Mortality Statistics: cause 1984. Series DH2, No. 11. HMSO, London.

ROBERTS, M.M., ALEXANDER, F.E., ANDERSON, T.J. & 9 others (1990). Edinburgh trial of screening for breast cancer: mortality at seven years. *Lancet*, **335**, 241.

SHAPIRO, S., VENET, W., STRAX, P. & VENET, L. (1988). Periodic Screening for Breast Cancer. The Health Insurance Plan Project and Its Sequelae, 1963–1988. The John Hopkins University Press, Baltimore & London.

TABAR, L., GAD, A., HOLMBERG, L.H. & 6 others (1985). Reduction in mortality from breast cancer after mass screening with mammography. *Lancet*, **i**, 829.

TABAR, L., FAGERBERG, G., DUFFY, S.W. & DAY, N.E. (1989). The Swedish two county trial of mammographic screening for breast cancer: recent results and calculation of benefit. *J. Epid. & Comm. Hlth.*, **43**, 107.

UK TRIAL OF EARLY DETECTION OF BREAST CANCER GROUP (1981). The trial of Early Detection of Breast Cancer: description of method. *Br. J. Cancer*, **44**, 618.

UK TRIAL OF EARLY DETECTION OF BREAST CANCER GROUP (1988). First results on mortality reduction in the UK Trial of Early Detection of Breast Cancer. *Lancet*, **ii**, 411.

WAALER, E. & GRIMSTEAD, M. (1958). The clinical diagnoses of the cause of death and their reliability. *Acta Path. & Microb. Scand.*, **43**, 330.

WALDRON, H. & VICKERSTAFF, L. (1977). Intimitations of Quality. London Nuffield Foundation Hospital Trust.